# Annealing Effect on the Structural and Optical Properties of Sputter-Grown Bismuth Titanium Oxide Thin Films

**DOI:** 10.3390/ma7053427

**Published:** 2014-04-30

**Authors:** José E. Alfonso, Jhon J. Olaya, Claudia M. Bedoya-Hincapié, Johann Toudert, Rosalia Serna

**Affiliations:** 1 Grupo de ciencia de materiales y superficies, Universidad Nacional de Colombia, Bogotá AA 14490, Colombia; E-Mails: jjolayaf@unal.edu.co (J.J.O.); cbedoyah@unal.edu.co (C.M.B.-H.); 2 Centro Internacional de Física (CIF), Bogotá AA 14490, Colombia; 3 Grupo de Análisis de Fallas, Integridad y Superficies (AFIS), Universidad Nacional de Colombia, Bogotá AA 14490, Colombia; 4 Physics, Chemistry, Mathematics Computational Applications (PCM), Universidad Nacional de Colombia, Manizales AA 127, Colombia; 5 Laser Processing Group, Instituto de Optica, CSIC, c/Serrano 121, Madrid 28006, Spain; E-Mails: johann.toudert@gmail.com (J.T.); rosalia.serna@csic.es (R.S.)

**Keywords:** thin films, optical characterization, refractive index

## Abstract

The aim of this work is to assess the evolution of the structural and optical properties of Bi*_x_*Ti*_y_*O*_z_* films grown by rf magnetron sputtering upon post-deposition annealing treatments in order to obtain good quality films with large grain size, low defect density and high refractive index similar to that of single crystals. Films with thickness in the range of 220–250 nm have been successfully grown. After annealing treatment at 600 °C the films show excellent transparency and full crystallization. It is shown that to achieve larger crystallite sizes, up to 17 nm, it is better to carry the annealing under dry air than under oxygen atmosphere, probably because the nucleation rate is reduced. The refractive index of the films is similar under both atmospheres and it is very high (*n* =2.5 at 589 nm). However it is still slightly lower than that of the single crystal value due to the polycrystalline morphology of the thin films.

## 1. Introduction

Bismuth titanium oxide is of interest because it has several applications in the field of microelectronics, electro-optics and dielectrics devices. Numerous compounds of the Bi-Ti-O system are known, such as: Bi_4_Ti_3_O_12_, Bi_2_T_i2_O_7_, Bi_2_Ti_4_O_11_, Bi_12_TiO_20_ and Bi_20_TiO_32_ [1]. The phase Bi_4_Ti_3_O_12_ (BIT), when it crystallizes in the monoclinic or orthorhombic phase, exhibits ferroelectric properties that have attracted much attention due to its applications in the electronic industry as capacitors and high temperature piezoelectric devices [2,3], besides being regarded as one of the most promising candidate materials for nonvolatile ferroelectric memories owing to its large spontaneous polarization and its fatigue-resistance property [4–7]. Moreover, the possibility of fabricating environmentally friendly electrical devices without lead is an outstanding reason for synthesizing the BIT compound [8]. This stoichiometric can be obtained through thermal treatments of the Bi_12_TiO_20_ compound, which shows photocatalitic activity [9]. Additionally, the BIT compound also has applications in electro-optic devices, but their optical properties, such as the refractive index, have barely been studied. However, there are published papers that give account of the techniques employed to grow BTO films, such as: metal organic solution [10], chemical solution deposition [11], and magnetron sputtering [12]. In some previous works it has been shown that annealing treatments can change the optical properties of the films; however there is no detailed study on the grain size evolution and its effect on the optical properties. In this work we study in detail the effect of the annealing treatments both on the microstructure and optical properties of BiTiO thin films grown by sputtering.

## 2. Results and Discussion

**Figure 1.**
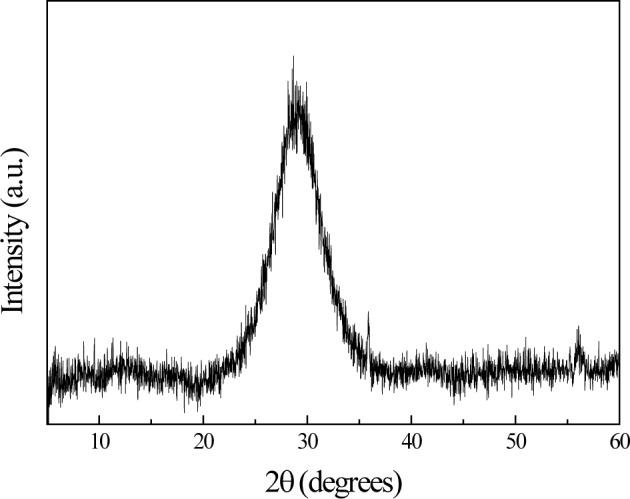
XRD pattern of bismuth titanium oxide thin film without annealing treatment.

The XRD pattern of the as-grown thin film (*i.e*., at 350 °C) showed be amorphous, likely due to the fact that the Bi_12_TiO_20_ phase formation usually occurs between 350 and 550 °C [13], as it is shown in Figure 1.

**Figure 2.**
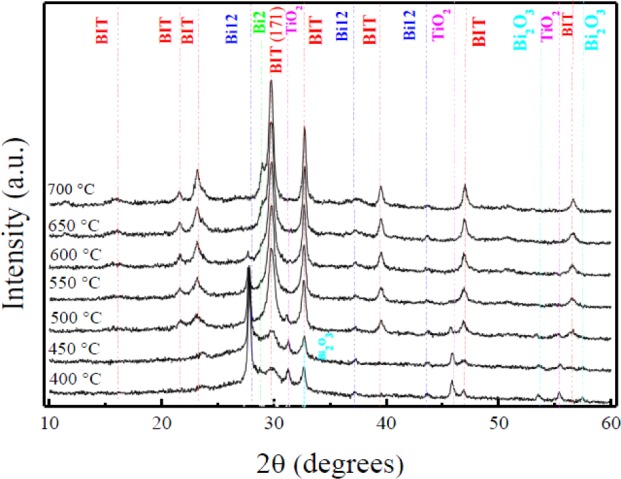
XRD patterns of Bismuth titanium oxide film with annealing treatment during 5 min in oxygen atmosphere. Bi*_x_*Ti*_y_*O*_z_* phases: Bi_4_Ti_3_O_12_ (BIT), Bi_12_TiO_20_ (Bi12), Bi_2_Ti_2_O_7_ (Bi_2_).

Figure 2 shows the effect of the annealing temperature on the crystalline structure of the thin films. At temperatures below 500 °C, the films mainly exhibit the crystallographic phase Bi_12_TiO_20_ (denoted as Bi12), with lower contributions of the Bi_4_Ti_3_O_12_, TiO_2_, and Bi_2_O_3_ phases. As the temperature increases, a gradual decrease of the Bi_12_TiO_20_, TiO_2_, and Bi_2_O_3_ phases is observed, and the notable start of the Bi_4_Ti_3_O_12_ formation was found around 500 °C [14]. These results are in agreement with those found by other authors, who have observed the formation of Bi_4_Ti_3_O_12_ between 500 and 700 °C [12]. However, the main peak of Bi_4_Ti_3_O_12_ indexed as (171) is slightly shifted toward left side with respect to its XRD powder, which is at 30.05° according to PDF card 35-0795. This shift is probably due to the stress between the substrate and the film. At temperatures above 600 °C, the Bi_2_Ti_2_O_7_ (denoted as Bi_2_) crystallographic phase is observed in the XRD pattern, and this is likely due to bismuth deficiency resulting from a bismuth loss because of re-sputtering phenomena, or through evaporation during the annealing treatment. This phase deficient in Bi appears together with the Bi_4_Ti_3_O_12_ crystallographic phase [15]. Additionally, in Figure 1 we can observe that as the temperature increases above 500 °C, the films are mainly composed of the Bi_4_Ti_3_O_12_ phase. The Full Width at Half Maximum (FWHM) along the (171) plane value decreases from 0.57° to 0.48° at 500° and 700 °C, respectively, exhibiting an improvement in crystallinity and an increase of the grain size [14]. After the above-described thermal analysis, additional annealing treatments of the films in controlled atmospheres of dry air and oxygen were performed at 600 °C. After these treatments, the films showed an enhanced crystallinity, and the presence of secondary crystallographic phases was minimal.

**Figure 3.**
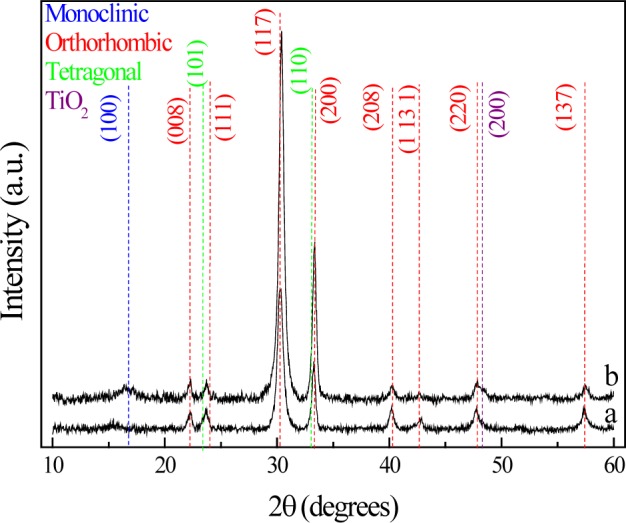
XRD patterns of bismuth titanium oxide thin films with annealing treatment in (**a**) oxygen; and (**b**) dry air atmospheres.

Figure 3 shows the XRD pattern of the films after 2 h of annealing at 600 °C in air and oxygen atmospheres. The XRD pattern shows that three different crystallographic structures of Bi_4_Ti_3_O_12_ have been formed: monoclinic, orthorhombic, and tetragonal, originating from the starting Bi_12_TiO_20_ during the thermal process [16].

**Figure 4.**
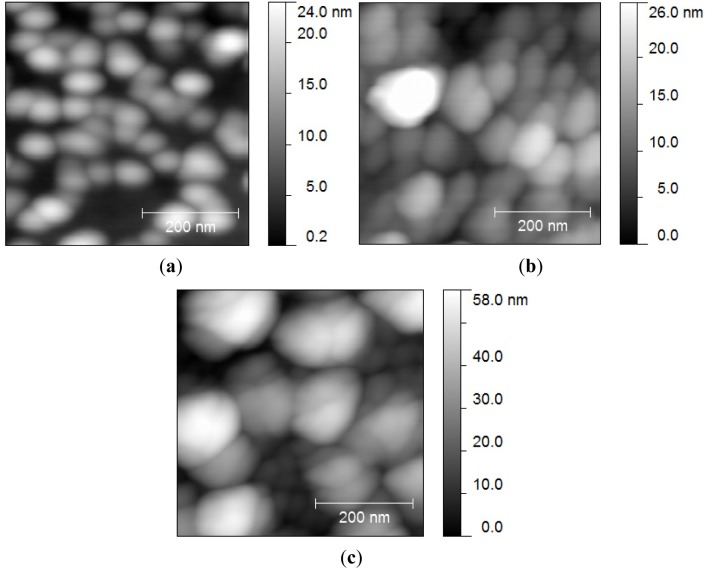
AFM images of: (**a**) as-grown thin films; (**b**) annealed films in oxygen and (**c**) annealed films in dry air.

The morphological behaviour of the thin films is showed in Figure 4. The as-grown film exhibited the lowest grain size of 3.4 nm and root mean square roughness (*rms)* of 4.3 ± 0.7 nm, while these values increased more in dry air than in oxygen by shifting the *rms* from 5.0 ± 1.8 to 13.7 ± 2.7, and the grain size from 9 to 17 nm, in oxygen and dry air atmospheres, respectively. This behaviour can be explained because of a higher rate of nucleation in oxygen than in air, leading to lower roughness and grain size values in this atmosphere [17]. The best-fit film thickness obtained from the ellipsometry modeling was 220 nm. This value is slightly lower to that obtained by perfilometry where the thickness of the films grown in the different atmospheres show average values of 250 nm.

**Figure 5.**
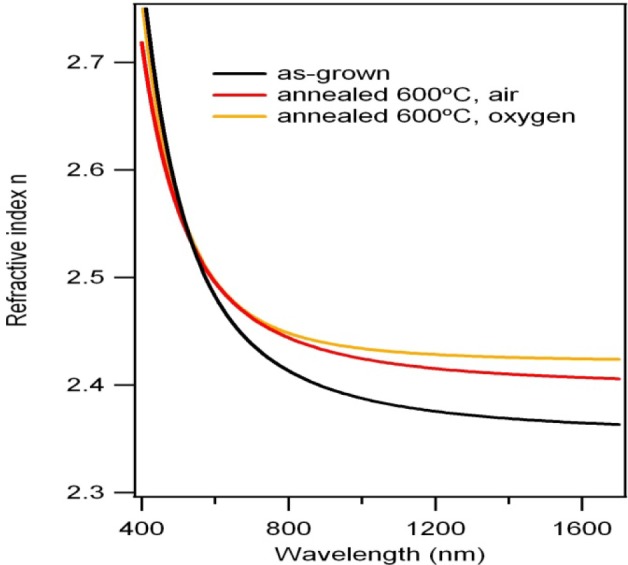
Best fit for the *n* spectra obtained for the three films using an isotropic model; analysis range 20°–60° and 300–1700 nm.

Figure 5 shows the best-fit spectrum of the refractive index *n* in the 400–1700 nm ranges for the as-grown and annealed films (at 600°C in oxygen and dry air atmosphere). Excellent multi-angle fitting (mean square errors around 3) was obtained along the whole wavelength range for all the films using a real Cauchy law (*k* = 0), showing that they exhibit high transparency in the visible and near-infrared ranges. From Figure 4 it can be seen that the refractive index increases with the annealing process in both atmospheres, at long wavelengths and don’t have important changes at short wavelengths, with respect to the values of refractive index of the as-grown film. These results are originating from the fact that high annealing temperature increases the crystal size of the film [10]. While the AFM results showed that the film annealed in dry air shows bigger grains than that annealed in oxygen, however index of refraction is higher in the last film, probably due that is more compact (see Figure 4) [10].

The optical properties of Bi_4_Ti_3_O_12_ thin films on Si (100) deposited at 600 °C from a sputtering gas mixture of 60% Ar and 40% O_2_ (without annealing treatment) were also studied by Yamaguchi *et al*. via ellipsometry [12]. Their refractive index showed a lower value at 700 nm of *n* ~ 2.26 nm, but additionally the thin film exhibits the BIT phase.

On the other hand, a comparative study of the optical properties of Bi_4_Ti_3_O_12_ and Bi_3.25_In_0.75_Ti_3_O_12_ thin films on quartz substrates was performed by Jia *et al*., using optical transmittance measurements [11]. At 589 nm, the Bi_4_Ti_3_O_12_ film of their paper exhibits a refractive index of 2.46, very close to our as-grown and annealed thin films (Figure 4, *n* ~ 2.5 at 589 nm) values. These values are still lower than those reported for a Bi_4_Ti_3_O_12_ single crystal (*n* = 2.7 at 589 nm [18]). Usually, the lower refractive index obtained for the thin films has been attributed to the smaller packing density and the higher density of defects in the films compared to that in the single crystals. Since the films are not single crystals, but are composed of small crystallites, it is likely that they are not as dense as the bulk material. So it is reasonable that the refractive indices of the thin films are lower, although at higher wavelengths, the refractive index tends to diminish, and a slight improvement in oxygen atmosphere can indicate a thin film with a more uniform surface than in dry air.

## 3. Experimental Details

The equipment used in the deposition of the bismuth titanium oxide films was a CIT-Alcatel HS 2000 rf sputtering system with a balanced magnetron 101.6 mm in diameter, described in a previous paper [19]. The deposition was performed from a target of Bi_4_Ti_3_O_12_ with high purity (99.9%) onto silicon (100) substrates. The conditions established for deposition were: target power supply 150 W, argon flux 20 sccm, deposition temperature 350 °C and deposition time 30 min. In order to study the influence of the annealing treatment on the crystalline structure and optical properties of the films, an annealing process was carried out on some of them by using a Lindberg Blue M furnace to obtain thin films annealed at 600°C for 2 h in dry air (19.9% O_2_) and in an oxygen atmosphere with a 20 sccm flux. In this way, three kinds of thin films were studied: as**-**grown, and with an annealing process in oxygen and in dry air atmospheres. The crystalline structure of these films (as-grown and annealed at 600 °C) was characterized with a Panalytical X’Pert PRO X Ray difractometer (XRD) using a Bragg Brentano geometry with Cu-kα radiation (λ = 1.5405 Å) in the 2θ range 10°–60° with steps of 0.02°. In addition, using the same equipment, the XRD patterns of an initially as-grown film was recorded *in-situ* as a function of the annealing temperature, which was increased from 400 to 700 °C, with steps of 50 °C, in a controlled oxygen atmosphere for 5 min and a 20 sccm flux. The thicknesses of the films were measurement with a Veeco Dektak 150 surface profilometer. Morphology response of all the films was measured with the Atomic Force Microscopy (AFM) Nanoscope IV Dimension 3100 equipment of Digital Instruments–Bruker Ellipsometry measurements on the bismuth titanium oxide thin films (as-grown and annealed at 600 °C) were performed from 20° to 60° (step 10°) in the 400–1700 nm wavelength range (step 10 nm) using a Woollam VASE vertical ellipsometer (J.A. Woollam, Lincoln, NE, USA). Fitting of the experimental data was achieved using a three-layer structure (roughness/film/substrate). The substrate shows the properties of bulk Si. The refractive index *n* of the films was modelled using a Cauchy function with no absorption (extinction coefficient *k* = 0), and the surface roughness layer was modelled using a standard effective medium approximation (EMA,) function implemented in the Woollam WVASE software The EMA used is the Bruggeman’s model assuming 50% material and 50% void for the surface layer refractive index. The roughness of the films obtained from the ellipsometry measurements was about 6 ± 2 nm which is in good agreement with the *rms* values obtained from the AFM measurements. The small differences could be due to the fact that AFM measurements are local, where in the optical measurements an average from a larger area (mm^2^) is obtained. A multi-angle fitting procedure was used, *i.e*., the same model was used to simultaneously fit the spectra obtained at the 5 different angles. The free parameters were the layer thicknesses and the Cauchy parameters (A*_n_*, B*_n_*, C*_n_*), and the thickness in homogeneity, which was always below 10%.

## 4. Conclusions

The structural and optical properties of Bi*_x_*Ti*_y_*O*_z_* thin films grown through rf magnetron sputtering as a function of thermal treatment were studied. The XRD results show that the as-grown films are amorphous. After annealing treatments, the presence of various Bi*_x_*Ti*_y_*O*_z_* crystalline phases and a lower concentration of oxide compounds (TiO_2_, Bi_2_O_3_) were identified. Optically, the thin films showed good transparency. The refractive index of the films increased after the annealing treatments in the near-infrared region, reaching a value as high as *n* ~ 2.45 at 850 nm, which can be attributed to the increase of the grain size with the thermal treatment. Basically, these results allowed us to establish relationships among crystallinity, morphology and optical properties of BiTiO films.

## References

[1] Zhang Y., Wang H., Shang S.X., Xu X.H., Yang X.N., Liu W.M. (2004). X-ray photoelectron spectroscopy study of La-modified Bi_2_Ti_2_O_7_ thin film. Mater. Lett.

[2] Ikegami S., Ueda I. (1974). Piezoelectricity in ceramics of ferroelectric bismuth compound with layer structure. Jpn. J. Appl. Phys.

[3] Takenaka T., Sakata K. (1984). Grain orientation effects on electrical properties of bismuth layer-structured ferroelectric Pb_1−_*_x_*(Na,Ce)*_x_*_/2_Bi_4_Ti_4_O_15_ solid solution. Jpn. J. Appl. Phys.

[4] Junquera J., Ghosez P. (2003). Critical thickness for ferroelectricity in perovskite ultrathin films. Nature.

[5] Stachiotti M.G., Rodriguez C.O., Ambrosch-Draxl C., Christensen N.E. (2000). Electronic structure and ferroelectricity in SrBi_2_Ta_2_O_9_. Phys. Rev. B.

[6] Paz de Araujo C.A., Cuchlaro J.D., McMillan L.D., Scott M.C., Scoot J.F. (1995). Fatigue-free ferroelectric capacitors with platinum electrodes. Nature.

[7] Miura K. (2002). Electronic properties of ferroelectric SrBi_2_Ta_2_O_9_, SrBi_2_Nb_2_O_9_, and PbBi_2_Nb_2_O_9_ with optimized structures. Appl. Lett.

[8] Stojanovic B.D., Simoes A.Z., Paiva-Santos C.O., Quinelato C., Longo E., Varela J.A. (2006). Effect of processing route on the phase formation and properties of Bi_4_Ti_3_O_12_ ceramics. Ceram. Int.

[9] Yao W.F., Wang H., Xu X.H., Cheng X.F., Huang J., Shang S.X., Yang X.N., Wang M. (2003). Photocatalytic property of bismuth titanate Bi_12_TiO_20_ crystals. Appl. Catal. A Gen.

[10] Wang X.-S., Zhai J.-W., Zhang L.-Y., Yao X. (1999). Structural and optical characterization of Bi_4_Ti_3_O_12_ thin films prepared by metallorganic solution deposition technique. Infrared Phys. Technol.

[11] Jia C., Chen Y., Ding L., Zhang W. (2007). Effect of incorporating nonlanthanoidal indium on optical properties of ferroelectric Bi_4_Ti_3_O_12_ thin films. Appl. Surf. Sci.

[12] Yamaguchi M., Nagamoto T., Omoto O. (1997). Preparation of highly *c*-axis-oriented Bi_4_Ti_3_O_12_ thin films and their crystallographic, dielectric and optical properties. Thin Solid Films.

[13] Liu W., Wang X., Tian D., Xiao C., Wei Z., Chen S. (2010). chemical reaction and crystalline procedure of bismuth titanate nanoparticles derived by metalorganic decomposition technique. Mater. Sci. Appl.

[14] Keeney L., Zhang P.F., Groh C., Pemble M.E., Whatmore R.W. (2010). Piezoresponse force microscopy investigations of Aurivillius phase thin films. J. Appl. Phys.

[15] Fu C., Huang J., Li J., Guo D. (2009). Effect of Bi content in precursor solutions on microstructure and ferroelectric properties of bismuth cerium titanate thin films. Sci. China Ser. E.

[16] Morozov M.I., Mezentseva L.P., Gusarov V.V. (2002). Mechanism of formation of Bi_4_Ti_3_O_12_. Russ. J. Gen. Chem.

[17] Simões A.Z., Ries A., Stojanovic B.D., Biasotto G., Longo E., Varela J. (2007). Electrical properties of lanthanum doped Bi_4_Ti_3_O_12_ thin films annealed in different atmospheres. Ceram. Int.

[18] Cummins S.E., Cross L.E. (1968). Electrical and optical properties of ferroelectric Bi_4_Ti_3_O_12_ single crystals. J. Appl. Phys.

[19] Alfonso J.E., Torres J., Marco J.F. (2006). Influence of the substrate bias voltage on the crystallographic structure and surface composition of Ti_6_Al_4_V thin films deposited by rf magnetron sputtering. Br. J. Phys.

